# Early immune responses and development of pathogenesis of avian infectious bronchitis viruses with different virulence profiles

**DOI:** 10.1371/journal.pone.0172275

**Published:** 2017-02-15

**Authors:** Cintia Hiromi Okino, Marcos Antônio Zanella Mores, Iara Maria Trevisol, Arlei Coldebella, Hélio José Montassier, Liana Brentano

**Affiliations:** 1 Embrapa Swine and Poultry, Concórdia, SC, Brazil; 2 Laboratory of Immunology and Virology (Imunovir), Department of Veterinary Pathology, Universidade Estadual Paulista—UNESP, Jaboticabal, SP, Brazil; University of Hong Kong, HONG KONG

## Abstract

Avian infectious bronchitis virus (IBV) primarily replicates in epithelial cells of the upper respiratory tract of chickens, inducing both morphological and immune modulatory changes. However, the association between the local immune responses induced by IBV and the mechanisms of pathogenesis has not yet been completely elucidated. This study compared the expression profile of genes related to immune responses in tracheal samples after challenge with two Brazilian field isolates (A and B) of IBV from the same genotype, associating these responses with viral replication and with pathological changes in trachea and kidney. We detected a suppressive effect on the early activation of TLR7 pathway, followed by lower expression levels of inflammatory related genes induced by challenge with the IBV B isolate when compared to the challenge with to the IBV A isolate. Cell-mediated immune (CMI) related genes presented also lower levels of expression in tracheal samples from birds challenged with B isolate at 1dpi. Increased viral load and a higher percentage of birds with relevant lesions were observed in both tracheal and renal samples from chickens exposed to challenge with IBV B isolate. This differential pattern of early immune responses developed after challenge with IBV B isolate, related to the downregulation of TLR7, leading to insufficient pro-inflammatory response and lower CMI responses, seem to have an association with a most severe renal lesion and an enhanced capability of replication of this isolate in chicken.

## Introduction

Avian infectious bronchitis virus (IBV) is a highly infectious causative agent of avian infectious bronchitis (IB), a disease of high economic impact, which affects poultry worldwide. IBV replicates primarily in tracheal epithelial cells, inducing several mucosal pathological changes, including ciliary loss, degeneration and necrosis of epithelial cells, glandular degeneration, inflammatory cell infiltration and epithelial hyperplasia [1,2]. After replication at primary site in tracheal mucosa, viraemia and IBV secondary replication are also found in other respiratory tissues (nose, lungs and air sacs) and in many non-respiratory epithelial tissues (kidneys, testes, oviduct, and gastrointestinal tract). Nephritis is commonly observed, mainly in broilers, and depending on the pathotype of the IBV strain and on the bird age, it may cause high mortality, and microscopic lesions of degeneration and necrosis of the renal tubular cells and interstitial inflammatory cell infiltrate [3,4,5,6].

Prevention of IBV infection is currently achieved through vaccination, especially by attenuated viral vaccines, suggesting that local mucosal immunity is essential for induction of effective protection against disease [7,8,9]. In a previous study, we demonstrated that the dose of attenuated vaccine administered by local route is closely related to humoral and cellular immune responses at tracheal mucosal sites and their ability to confer effective protection against disease [8].

The continuous emergence of new IBV variants in several countries [3,10,11,12,13] are routinely pointed out as a cause of outbreaks in vaccinated flocks, leading to significant economic losses to the poultry industry [14].

Despite the great number of IBV strains and variants described over the last years [14], most of the studies have been limited to the classification and differentiation of IBV strains in genotypes, pathotypes, protectotypes, and/or serotypes, while, the specific immune mechanisms involved in pathogenesis of this virus remains poorly elucidated.

A recent study has observed differential early immune response after infection with IBV when comparing IBV-susceptible and IBV-resistant chicken lines. Even before infection, specific genes, including genes related to innate immune response, were found to be differentially expressed in each chicken line. Despite these differences, viral loads were similar in tracheal samples of both chicken lines, indicating that IBV resistance might be associated with how the birds respond to the virus and not how they can prevent an initial infection [15].

The innate immune response is the first line of host defense against infections, and several immune and non-immune cells on mucosal surfaces are involved on recognition of pathogen associated molecular patterns (PAMP) of microorganisms. PAMPs are recognized through pattern recognition receptors (PRR), among which toll-like receptors (TLR) are an important group. During a RNA-virus infection, activation of innate immune responses depends on the interaction of endosome-associated (TLR3 and TLR7) and/or cytosol-located (RIG-I like receptors) PRRs. TLR3 and TLR7 interact with double-stranded (ds) RNA or single-strand (ss) RNA, respectively, and the subsequent activation pathways for TLR3 and TLR7 are mediated by TRIF (TIR-domain-containing adapter-inducing interferon-β) and MyD88 (myeloid differentiation primary response gene 88), respectively, which lead to the production of anti-viral type I interferons (IFNα and IFNβ) and pro-inflammatory cytokines (IL1β and IL6) [16]. Expression of genes related to the TLR pathway activation and type I IFN were already found to be upregulated in tracheal samples from birds challenged with reference strains of IBV [7,16,17].

The inducible nitric oxide synthase (iNOS or NOS2), responsible for the production of nitric oxide (NO) by macrophages in response against microbial infections, is often associated with synergic effects combined with acute phase proteins and cytokines, as IFNs and TNFs, leading to enhanced phagocytosis. Alternatively, an excessive production of iNOS and consequently NO may have negative effects by promoting excessive inflammation or apoptosis [18,19]. Although a previous report did not identify a change in iNOS transcripts in both tracheal and lung samples during the early phase of IBV infection [16], increased levels of this enzyme was associated with high degree of disease severity in chickens infected with avian influenza H5N1 [18], and virulent Newcastle disease [20].

The important role of pro-inflammatory cytokines on IBV pathogenicity was demonstrated in a previous evaluation of the kinetics of the inflammatory and cell-mediated immune responses in tracheal mucosa of birds infected with the M41 strain of IBV. TNFSF15 and TGFβ transcripts were found to peak at 1 day post infection (dpi), followed by higher upregulation of IL6, IL1β and IFNγ. These increased levels of pro-inflammatory cytokines were associated to highest scores of lesions and viral load at 3dpi, whereas later, at 7dpi, highest increases in CD8 and Granzyme homolog A mRNA expression were detected and associated to a significant decrease in lesions and viral load [2].

It has been recognized that IL6 is associated with development of tissue damage in chickens challenged with IBV, including nephritis [21]. Levels 20 times higher of IL6 mRNA after IBV challenge were observed in an IBV-susceptible chicken line compared to the disease-resistant chicken line [22]. High level of IL6 gene expression were detected in renal samples of chickens challenged with a nephropathogenic IBV strain and it has been well correlated with viral load and influx of inflammatory cells in this organ [6].

During the development of adaptive immunity against IBV infection, the local cell-mediated immune (CMI) response was associated with CD3+, CD8+ and CD4+ cells influx in the trachea from 3 to 7 dpi with IBV [1,9]. Furthermore, CD8 and other CMI related genes were markedly increased during secondary response related to memory protection induced by the first antigen IBV exposition at 1dpi (8). Conversely, the CMI genes were found most upregulated later, at 5dpi, in the primary adaptive immune response of non-vaccinated chickens and correlated with high virus load in trachea, indicating this response might be more involved in the IBV pathogenesis in this post-infection period of non-immune chickens [8].

Mechanisms underlying immune-pathogenesis caused by IBV and those relating to viral clearance have begun to be elucidated [2,4,7,8,16,17,22,23]. However, there are no studies regarding the existence of differential pathological and immunological profiles induced by IBV strains or isolates differing in virulence for chickens.

The aim of this study was to investigate and compare the pathogenesis and the innate and adaptive immune responses induced by the infection with two Brazilian field isolates of IBV classified in the BR-I genotype.

## Material and methods

### Virus

Two Brazilian field isolates BI/BR/Embrapa/331/2000 (accession number: KU727196, identified as IBV A isolate) and BI/BR/Embrapa/127/2006 (accession number: KU727200, identified as IBV B isolate), were isolated from clinical cases of avian infectious bronchitis in poultry flocks located in southern Brazil. Virus isolation and titration were carried out by inoculation of five 10-day-old specific pathogen-free (SPF) embryonated chicken eggs/sample via the allantoic sac route [24] and virus titers were expressed as 50% embryo infectious doses (EID_50_) according to the Reed & Muench method [25]. IBV field strains were genotyped by automated DNA sequencing (ABI3130XL, Applied Biosystems) of a 1059 bp portion of the S1 gene amplified as described [26]. The deduced amino acid sequences of S1 protein of IBV A and B isolates have 93.16% of identity, and with regard H120 strain (Massachusetts serotype) of IBV they have 69.4% and 70.0% identity, respectively.

### Ethics statement

The chickens were housed and handled at the Laboratory of Animal Health and Genetics—Embrapa Swine and Poultry, Concórdia- SC, Brazil. All procedures have been approved by the Embrapa Swine and Poultry Ethical Committee for Animal Experimentation (CEUA/CNPSA), in accordance with ethical principles and guidelines of animal experimentation adopted by the Brazilian College of Experimentation (process number 016/2012).

### Experimental design

Three groups of Specific Pathogen Free (SPF) chicks (White Leghorn lineage) obtained from Valo Biomedia (Uberlândia, SP, Brazil) were housed in separate positive pressure isolators. At 28 days of age, groups A and B were respectively challenged with 10^4.0^ EID_50_/bird of A strain of IBV and 10^4.0^ EID_50_/bird of B strain of IBV by intra-ocular and intra-nasal routes. One group remained mock infected (negative control group, referred as NC). During whole experimentation, chickens were monitored twice a day. If animals have presented severe depression and lethargy, they were separated to be euthanized, though, there were no animals in these conditions during experiment. The birds from groups A, B and NC were randomly euthanized by cervical dislocation, at 1, 5 and 8 day post-infection (dpi). Tracheal and renal samples were collected from each group; a portion of the proximal third from each tracheal sample and a fragment of distal left kidney were immediately frozen in liquid nitrogen and kept at -70°C until processing for RNA extraction. Another portion of tracheal and renal samples were subjected to histopathological analysis. Throughout the experimental period, birds received water and feed “ad libitum”, the room temperature was adapted according with the bird´s age, and the monitored ammonia concentration levels remained below 2 parts per million (ppm).

### Microscopic pathological alterations

Tracheal samples were divided into three fragments (proximal, medial and distal), and a portion of each fragment was prepared for histopathology. The severity of observed lesions scores were determined as previously described [8]. Absence of injury was classified as 0, while mild, moderate and severe were scored as grades 1 to 3. Morphological characteristics observed by histopathology included loss of cilia and epithelial cells, degeneration or necrosis of epithelial cells, degeneration of mucous glands, mucosal inflammatory infiltrate and epithelial hyperplasia. A sum of all scores of microscopic lesions from all evaluated fragments was amounted to final score per bird, ranging from 0 to 45. Birds presenting score values ≥ 2 in two or more evaluated parameters or presenting marked heterophilic infiltration were considered as relevant lesions induced by IBV.

The scores of microscopic lesions in the kidney samples were determined as previously described [6]. The parameters evaluated included tubular degeneration and necrosis, and presence of inflammatory infiltrate, and the scores ranged from 0 to 3 according to the severity of lesions.

### Real-time quantitative reverse transcription-polymerase chain reaction (RT-qPCR)

RNA extractions from the proximal third of tracheal and renal samples of experimentally infected chickens were performed using TRIzol Reagent (Invitrogen) followed by RNA purification using RNeasy Mini Kit (Qiagen). The extracted RNA purity and quantification were estimated by 260nm ultraviolet absorbance and readings at 260/280nm, respectively. RNA quality was verified with Agilent RNA 6000 Nano Kit (Agilent Technologies) in an Agilent 2100 Bioanalyzer instrument (Agilent Technologies) for determination of RIN (RNA Integrity Number) or by RNA analysis in 1% gel electrophoresis.

All tracheal and renal samples were tested for IBV viral load by RT-qPCR (hydrolysis probe system) using AgPath-ID One step RT-PCR kit (Ambion), primers and LNA-probe (5´FAM-3´BHQ1, IDT) for amplification of the 3´UTR genome region of IBV, as described [27]. Cq (Cycle quantification) results were used to calculate the Log of RNA copies (Log10) using the linear equation from a standard curve. Samples presenting Cq ≤ 36 were classified as positive for IBV.

Two-step RT-qPCR was used for the relative quantification of gene expression in the tracheal samples. cDNAs were synthesised according to instructions provided with High Capacity cDNA Reverse Transcription kit (Applied Biosystems) and 1μL (500 ng/μL) of Oligo(dT) primers (IDT). The PCR reactions contained 20 ng of cDNA, 7.5 μL of 2X Quantifast SYBR Green Master Mix (Qiagen), and 3 μM of each primer (Table 1) in a final volume of 15 μL. Amplification included a pre-incubation step at 95°C for 5 min, followed by 40 cycles of 95°C for 15 seconds and 60.0°C for 35 seconds. After amplification, a melting curve analysis was performed by raising the incubation temperature from 65°C to 95°C in 0.2°C increments with a hold step of 1 sec at each increment. All oligonucleotides used were designed using Primer3 [http://frodo.wi.mit.edu] software, spanning exons according to gene sequences from Ensembl [http://ensembl.org] and mRNA sequences deposited in GenBank. Except for IFNα and IFNβ which are intron-less mRNAs, then, residual genomic DNA was digested with 2 μL DNAse I (Promega) before cDNA synthesis. Efficiency of each specific Real-time PCR was calculated using two-fold serial dilutions of cDNA pooled from all tested animals, and Cq values plotted into the linear equation.

**Table 1:** Sequence of primers used in RT-qPCR for relative quantification of gene expression (immune responses and reference genes in chickens).

Gene	Primers (5´-3´)	Accession number	Location (nt)	Product size	Exon boundary	Efficiency	Y-int
IFNγ	Forward: AGCCGCACATCAAACACATA	NM_205149.1	374–488	115 bp	3/4	101.94	33.7428
	Reverse: AAGTCGTTCATCGGGAGCTT						
IL6	Forward: GTTCGCCTTTCAGACCTACCTG	NM_204628.1	442–571	130 bp	4/5	95.23	33.8663
	Reverse: ATCGGGATTTATCACCATCTGC						
INOS	Forward: ATTCTTATTGGCCCAGGAACAG	NM_204961.1	3156–3251	96 bp	25/26	86.05	30.9381
	Reverse: GTCACCACCTTTGATCCCTTTC						
CD4	Forward: CAAAAGTGGAGGTGAACGTCAG	NM_204649.1	1311–1416	106 bp	7/8	95.47	27.9984
	Reverse: ACATGAGCTTCCTCCACGGTAT						
CD3ε	Forward: GGGACCACAGTGACAATCACAT	NM_206904.1	211–394	184 bp	5/6	92.15	28.0915
	Reverse: AGTTTGCACACACTTTGGCATT						
HPRT1	Forward: GCCAGACTTTGTTGGATTTGAA	NM_204848.1	572–728	157 bp	5/6	83.64	32.958
	Reverse: AGCCATAGCACTTCAACTGTGC						
TOP2B	Forward: AAGGCCAAGAAGATGGAAACTG	NM_205082.1	4748–4934	187 bp	35/36	91.46	30.668
	Reverse: TCTTGGATTTCTTGCATGGTGT						
TLR3	Forward: TCCATGGTGCAGGAAGTTTAAG	NM_001011691.3	2448–2575	128 bp	3/4	92.95	26.1027
	Reverse: TCAAGCAAAGTGCATGATTCAA						
MYD88	Forward: AGAGTTGGAGCAAACGGAGTTC	NM_001030962.1	565–682	118 bp	3/4	94.58	30.708
	Reverse: CATCCTCCGACACCTTCTTTCT						
TLR7	Forward: TTGCTGCTGTTGTCTTGAGTGA	NM_001011688.2	49–213	165 bp	1/2	99.60	31.4145
	Reverse: CGTCCTTGCATGATGTACCATT						
IFNα	Forward: GTCTTGCTCCTTCAACGACACC	GU119896.1	198–295	98bp	intron-less	98.5975	31.589
	Reverse: TGAGGATTTTGAAGAGGTGCTG						
IFNβ	Forward: AAAGCAAGGACAAGAAGCAAGC	GU119897.1	215–353	139bp	intron-less	94.832	33.901
	Reverse: TAATGCTGGATCTGGTTGAGGA						
IL1β	Forward: CCTTCGACATCTTCGACATCAA	NM_204524.1	379–491	113 bp	4/5	105.83	33.5343
	Reverse: AATGTTGAGCCTCACTTTCTGG						
CD8β	Forward: CTGCATGGCTCCGACAATGG	NM_205247.2	710–802	93bp	2/3	92.4496	31.8788
	Reverse: ATCGACCACGTCAAGCTGGG						
Granzyme A	Forward: GCGTAGCAGGATGGGGACAA	NM_204457.1	429–627	199bp	4/5	104.46	34.5033
	Reverse: CCACCTGAATCCCCTCGACA						
TNFSF15	Forward: CTGCTGTTACAGACACGCTTCC	NM_001024578.1	263–341	79bp	2/3	98.3383	29.5086
	Reverse: GTGCTGGAGGGTTCTTGTTTCT						

The relative expression of all tested genes (Table 1) in tracheal samples of IBV-infected chickens was quantified as the fold change relative to the non-infected group (negative control), and the gene expression from each sample were standardised using the Cq value of the TOP2B/HPRT1 constitutive reference genes for the same sample [28]. The stability of the reference genes was tested using four candidates (TOP2B, HPRT1, GAPDH and Histone H3) and the best genes were selected using Bestkeeper and Normfinder softwares.

### Statistical analysis

The comparisons of relative changes in gene expression, viral load and microscopic lesions between the experimental groups were performed using the Kruskal-Wallis test followed by Wilcoxon test.

All analyses were conducted using the SAS 9.4 software (2012), and the probability level for significance was set as *p* ≤ 0.05.

## Results

### Microscopic pathological alterations in IBV infected chickens

**Fig 1:**
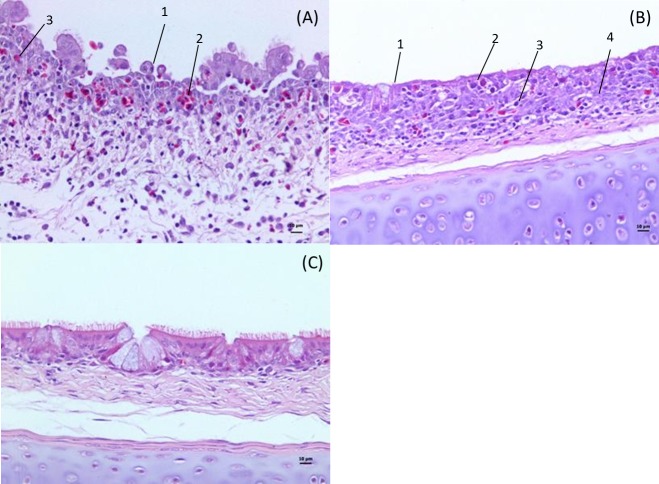
Micrographs showing tracheal histopathology from chickens challenged with IBV or mock infected. (A) Proximal third of trachea from the group challenged with IBV A strain, at 1dpi, showing acute tracheitis with epithelial cells losses [1], congestion [2], heterophilic cell infiltrate [3]. (B) Proximal third of trachea from the group challenged with IBV A strain, at 5dpi, showing ciliary loss [1], degeneration and necrosis of some epithelial cells [2], lymphoid cell infiltrate [3] and epithelial hyperplasia [4]. (C) Proximal third of trachea from NC group.

The tracheal lesions observed in groups challenged with A or B IBV isolates at 1 dpi were characterized by mild acute tracheitis, consisting of the presence of heterophils and mucus exudation in the tracheal lumen with congestion and heterophilic infiltration in the lamina propria and epithelial cell desquamation (Fig 1A). The most prominent lesions were observed at 5 dpi, with lesion scores ranging from 20 to 30, consisting of tracheitis. The microscopic pathological changes consisted mainly of lymphoplasmacytic inflammatory infiltrate in the mucosa, ciliary loss and epithelial hyperplasia (7 1B). Degeneration of the mucous glands and of epithelial cells was also observed at lower frequency and intensity. At 8 dpi, lesions were mild, tending to tissue recovery. Scores between 7 and 15 were found mainly in the challenged groups. The main histopathological changes were degeneration of the mucous glands, lymphocytic infiltration in the mucosa and epithelial hyperplasia. No relevant microscopic tracheal alterations were observed in the NC group at all post-infection intervals analyzed (Fig 1C).

**Fig 2:**
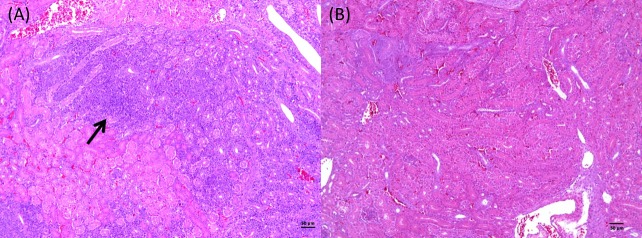
Micrographs showing kidney histopathology from chickens experimentally infected with IBV or mock infected. (A) Distal fragment of right kidney from the group challenged with IBV B strain, at 8dpi, presenting (arrow) marked lymphoplasmacytic intersticial nephritis (score 3). (B) Distal fragment of right kidney from the negative control group.

Marked lymphoplasmacytic interstitial nephritis (lesion score 3) was only observed in the challenged groups (A and B), at 8dpi (Fig 2A). Tubular necrosis was not observed in any renal sample from all experimental groups.

A higher percentage of birds challenged with IBV B isolate showed relevant tracheal (at 1dpi and 5dpi) and renal (at 8dpi) microscopic lesions (Table 2) compared to the birds challenge with IBV A isolate.

**Table 2:** Percentage of chickens experimentally infected with IBV or mock infected, with relevant tracheal and renal microscopic lesions, at 1dpi, 5dpi and 8dpi.

Group	Chickens with lesions at 1dpi*	Chickens with lesions at 5dpi*		Chickens with lesions at 8dpi*	
	Trachea	Trachea	Kidney	Trachea	Kidney
A	22.22% (2/9)	88.89% (8/9)	0% (0/9)	100% (6/6)	16.67% (1/6)
B	33.33% (3/9)	100% (9/9)	0% (0/9)	100% (5/5)	40% (2/5)
NC	0% (0/9)	0% (0/9)	0% (0/9)	0% (0/5)	0% (0/5)

NC = Negative control group; A = Group challenged with IBV A isolate; B = Group challenged with IBV B isolate.

* Percentage of chickens with relevant lesions and number of chickens with relevant lesions/total examined per group.

### Viral load in IBV infected chickens

**Fig 3:**
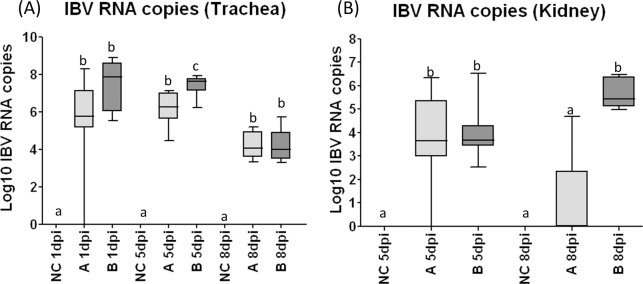
Log10 of IBV RNA copies in tracheas and kidneys from experimentally infected chickens or mock infected. (A) Log10 of IBV RNA copies in tracheas from chickens challenged with IBV A isolate and IBV B isolate, collected at 1dpi, 5dpi and 8dpi. (B) Log10 of IBV RNA copies in kidneys from chickens challenged with IBV A isolate and IBV B isolate, collected at 5dpi and 8dpi. Medians followed by different letters differ significantly (p≤0.05).

No positive samples were detected for the presence of IBV genome in tracheal and renal samples from the negative control (NC) group in any of the post-infection intervals analysed (Fig 3 and Table 3).

**Table 3:** Percentage of positivity for the presence of IBV genome in tracheal and renal samples from chickens experimentally infected with IBV A isolate, or IBV B isolate, or mock infected, at 1dpi (trachea), 5dpi and 8dpi (trachea and kidneys).

Group	IBV positive birds at 1dpi	IBV positive birds at 5dpi		IBV positive birds at 8dpi	
	Trachea	Trachea	Kidney	Trachea	Kidney
NC	0/8 (0%)*	0/9 (0%)	0/9 (0%)	0/5 (0%)	0/5 (0%)
A	7/8 (87.5%)*	9/9 (100%)	8/9 (88.89%)	6/6 (100%)	1/6 (16.67%)
B	9/9 (100%)	9/9 (100%)	9/9 (100%)	5/5 (100%)	5/5 (100%)

NC = Negative control group; A = Group challenged with IBV A isolate; B = Group challenged with IBV B isolate.

* One sample was lost during processing.

High percentages (87.5% and 100%) of tracheal samples were positive at 1 dpi for the presence of IBV genome from chickens challenged with IBV A and B strains, and differed significantly from tracheal samples from NC group (Fig 3A and Table 3). At 5dpi, all chickens from the challenged groups were IBV positive, with similar virus loads to those detected at 1dpi, differing also significantly different from NC group.

The renal samples from chickens of A group showed 88.89% of positivity for IBV genome in kidney samples while all the renal samples from chickens of B group were positive for IBV genome, (Fig 3 and Table 3). The presence and quantity of IBV genome in renal samples differed significantly from those of NC group.

**Fig 4:**
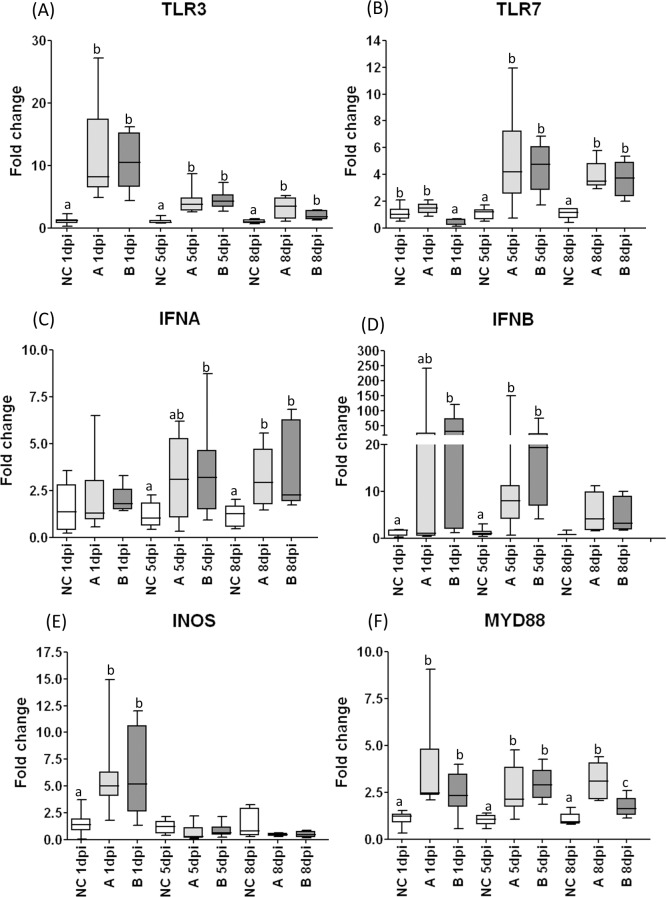
Relative expression of innate immune response related genes in tracheal samples from chickens infected with IBV. Relative expression of innate immune response related genes measured by RT-qPCR: TLR3 (A), TLR7 (B), IFNα (C), IFNβ (D), iNOS (E) and MyD88 (F), in tracheal samples collected 1 dpi, 5dpi and 8dpi from chickens experimentally challenged at 28 days of age with Brazilian field isolates A or B of infectious bronchitis virus, or mock infected (NC). Medians followed by different letters differ significantly (P≤0.05).

The viral load decreased at 8dpi in tracheal samples from all IBV challenged groups (A and B) (Fig 4B). Despite of lower virus levels, all challenged groups maintained 100% of positive birds (Table 3). Additionally, all the chickens from group challenged with IBV B isolate were positive for the presence of IBV genome in renal samples and only 16.67% were positive for the A challenged group, a significant higher viral load was found in B group when compared to other experimental groups (Fig 3B and Table 3).

Significantly higher means of IBV genome load (10 to 1000 times higher) were detected in group challenged with IBV B isolate compared to the group challenged with IBV A isolate, at 5dpi interval in tracheal samples (P = 0.0041) and at 8dpi in renal samples (P = 0.0041). (Fig 3).

### Innate immune responses in trachea

The mRNA expression data for TLR3, TLR7, MyD88, iNOS, IFNα and IFNβ in tracheal samples are illustrated in Fig 4. TLR3 was significantly upregulated and peaked at 1dpi in IBV challenged groups. At 5dpi, TLR3 transcripts also remained upregulated in challenged groups, although the levels were lower compared to values found at 1dpi. At 8dpi, TLR3 mRNA dropped markedly in challenged groups, but still remained significantly upregulated (Fig 4A). No significant differences were detected in the expression of TLR3 gene between the tracheal samples of chickens infected with A and B IBV isolates, and significant differences were seen by comparing the birds from groups A and B with those from control mock-infected birds.

TLR7 transcripts were downregulated (5 times) at 1 dpi only in the tracheal samples from B-challenged group, but their levels of expression rose at 5dpi and maintained at high levels at 8dpi in all challenged groups (A and B) (Fig 4B).

MyD88 mRNA transcripts were upregulated in tracheal samples from all challenged groups (A and B) and at all post-infection intervals. At 8dpi, the samples from A group presented higher levels than those from B group (P = 0.0176) (Fig 4F).

IFNα was upregulated at 5dpi and 8dpi in tracheal samples from chickens of B group, and only at 8dpi in the samples from A group chickens. IFNβ transcripts were upregulated in chickens from B group at 1dpi and 5dpi, while in chickens from the A group, only at 5dpi (Fig 4C and 4D).

The iNOS gene showed a significant increase in mRNA expression at 1dpi in challenged groups. At 5dpi and 8dpi, these transcripts dropped to basal levels (Fig 4E).

### Inflammatory responses in trachea

**Fig 5:**
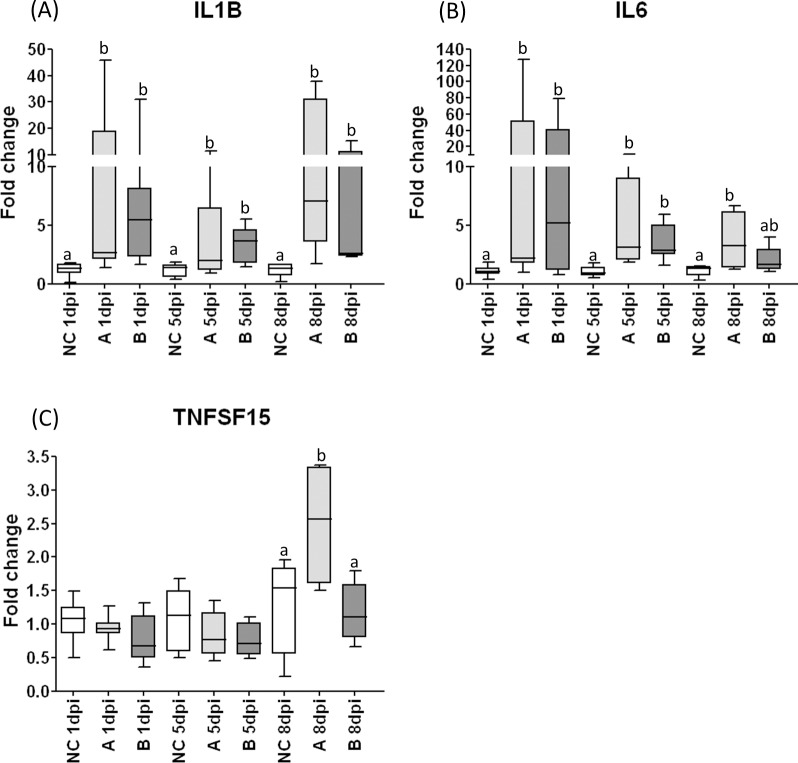
Relative expression of inflammatory response related genes in tracheal samples from chickens experimentally infected with IBV or mock infected. Relative expression of inflammatory response related genes measured by RT-qPCR: IL1β (A), IL6 (B) and TNFSF15 (C), in tracheal samples collected 1 day-post infection (dpi), 5dpi and 8dpi from chickens experimentally challenged at 28 days of age with Brazilian field isolates A or B of infectious bronchitis virus, or mock infected (control group). Medians followed by different letters differ significantly (p≤0.05).

IL1β transcripts were significantly upregulated in tracheal samples from all challenged groups at 1, 5 and 8 dpi and the highest levels of IL1β expression were reached at 8dpi. (Fig 5A).

IL6 mRNA transcripts were significantly upregulated in tracheal samples from challenged groups at 1 and 5 dpi. At 8 dpi, only the samples from group A showed upregulation in the expression of this cytokine gene (Fig 5B).

The TNFSF15 mRNA was only significantly upregulated at 8dpi, in the group challenged with IBV A isolate (Fig 5C).

### Cell-mediated immune responses in trachea

**Fig 6:**
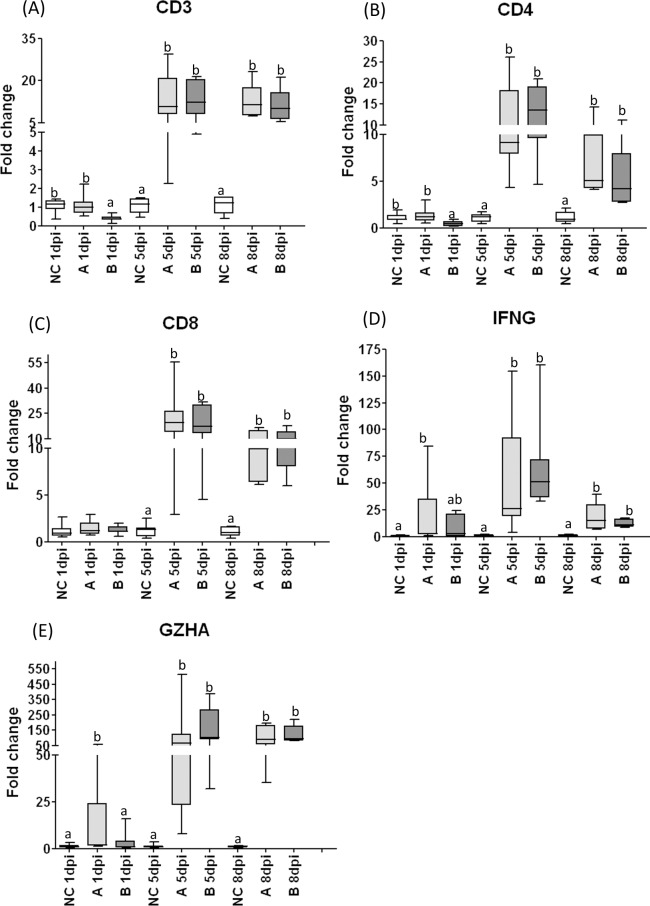
Relative expression of cell-mediated immune response related genes in tracheal samples from chickens experimentally infected with IBV or mock infected. Relative expression of cell-mediated immune response related genes measured by RT-qPCR: CD3 (A), CD4 (B), CD8β (C), IFNγ (D) and Granzyme homolog A (E), in tracheal samples collected 1 day-post infection (dpi), 5dpi and 8dpi from chickens experimentally challenged at 28 days of age with Brazilian field isolates A or B of infectious bronchitis virus, or mock infected (NC). Medians followed by different letters differ significantly (p≤0.05).

The CD3 and CD4 mRNA transcripts were downregulated at 1dpi only in the tracheal samples from the B group. At 5dpi and 8dpi, all tracheal samples from challenged groups showed upregulation of CD3 and CD4 expression, and the highest levels of expression of these genes were observed at 5dpi (Fig 6A and 6B).

At 5dpi and 8dpi, all challenged groups presented upregulation of CD8β transcripts, and the highest levels of expression of this gene were detected at 5dpi (Fig 6C).

The levels of IFNγ gene expression started to increase at 1dpi in group challenged with IBV A isolate. All challenged groups, presented upregulation of IFNγ transcripts, at 5dpi and 8dpi and the highest levels were found 5dpi (Fig 6D).

The transcripts of Granzyme homolog A mRNA were upregulated in all challenged groups at the three post-infection intervals evaluated, except for the group challenged with IBV B isolate, at 1dpi (Fig 6E).

## Discussion

The host-virus interactions that result in more severe pathogenesis induced by different pathotypes of IBV are not fully elucidate, but the IBV pathogenesis has been associated with high virus replication rates, induction of exacerbated inflammatory responses and delayed activation of anti-viral effector mechanism of innate and adaptive immune responses [2,16,31]. In this study, we found a suppressive effect on expression of some early innate and adaptive cell-mediated immune genes in the primary site of virus replication (trachea) from chickens infected with one of the tested IBV isolates (B). This possibly contributed to an exacerbated pathogenicity of this IBV isolate, especially for kidney tissues, but not for A isolate.

IBV B isolate demonstrated an enhanced ability for viral replication in chicken host, as we observed significantly higher IBV genome loads in tracheal samples (at 5dpi), and in kidneys (at 8dpi) of birds challenged with this isolate, compared to the group infected with IBV A isolate. Furthermore, a higher percentage of positive birds for IBV genome and the presence of pathological alterations were observed in the IBV B infected group, especially in renal samples, that were IBV positive in birds infected with this isolate at 8dpi, while only 17% were positive in the group infected with IBV A isolate.

This enhanced viral replication of IBV B isolate compared to A isolate, may have played a role as one of the determinants for the higher percentage of chickens with relevant microscopic tracheal and renal lesions in birds from the IBV B challenged group. This could result in increased pathology of the IBV B isolate. However, the reason for the differences observed in tissue tropism and pathogenicity of different IBV strains remains an open question and cannot be associated in the present study to differences in S1 protein sequences, as A and B IBV isolates have an identity of XX % for this protein. Similarly, a recent study demonstrated that despite the same efficiency of S1 protein binding to the epithelial cells of oviduct, in a sialic-acid dependent manner, observed for Qx IBV strain (associated with nephropathogenicity and reproductive tract disorders) and B1648 IBV strain (associated with nephropathogenicity), a high susceptibility to infection was observed for QX strain in contrast to the resistance to infection with B1648 strain [29]. The authors concluded that other cellular receptors and post-virus binding activation steps could be involved and play a role in the development of IB disease, although the attachment of IBV to host cells is considered as the first important step in virus infection and for determining the tissue tropism for this virus [29].

In view of the differences observed here for these two Brazilian IBV isolates (A and B) in terms of viral replication and pathological alterations induced, the current study investigated how innate and adaptive immune responses are developed after challenge with each one of these viruses, aiming to better understand the virus-host interactions and the putative immune-related mechanisms that might determine the course of pathogenicity induced by IBV isolates with different virulence profiles.

Several studies on innate immune responses elicited by viruses and mediated by PRRs have demonstrated that the levels of expression of TLR genes are usually upregulated, in order to activate intra-cellular pathways involved in the production of type I anti-viral IFNs and pro-inflammatory cytokines [30,7,16,17]. TLR7 was reported to be upregulated at 1dpi in trachea from chickens experimentally infected with a Connecticut strain of IBV, but then the expression of this gene decayed to basal levels and remained unaltered at 2dpi and 3dpi [16]. In contrast, in the current study an unexpected suppressive effect in the TLR7 gene expression was observed only during the early phase of IBV B isolate infection (1dpi), as the transcripts of this gene were found 5 times downregulated. Moreover, at 8dpi, other genes related to activation of TLR7 pathway were also affected, including the adapter MyD88 and the pro-inflammatory cytokine gene (TNFSF15), which showed lower levels of expression in the birds from the group challenged with B isolate compared to the group challenged with isolate A. Thus, these results demonstrate a differential profile of early type of innate immune responses induced by IBV B isolate compared to the IBV-induced innate immune responses of another study [16]. In the later study, an upregulation in the transcription of TLR7 and MyD88 genes was observed at 1dpi in tracheal samples from chickens infected with a Connecticut strain of IBV, that has a typical respiratory and pathogenicity for chicken respiratory tract.

Although other genes related to innate immune responses were also found to be differentially expressed in response to infection with the Brazilian IBV isolates, including TLR3, type I IFNs (IFNα and IFNβ) and iNOS, no significant differences were observed between the group infected with IBV B isolate and that infected with IBV A isolate. These results suggested that the identified differences between the two IBV isolates are not related to the biological activities associated with these innate response genes. However, TLR3 and iNOS genes were significantly upregulated and peaked at 1dpi, while type I IFNs were significantly upregulated and peaked at 5dpi. These results contradict other findings for TLR3, IFNα, IFNβ and iNOS gene expression in the respiratory tract of chickens challenged with a Connecticut strain of IBV [16], since no significant differences were observed for iNOS and IFNα gene expression, and TLR3 was unaltered at 1dpi and IFNβ was upregulated at 1dpi. This indicates the likely existence of others factors and pathways in the innate immune responses that are triggered by the interaction of host and different pathotypes of IBV as well possible differences in structural and non-structural proteins of IBV strains involved in virus evasion from the immune responses [31].

As memory immune responses induced by IBV vaccination could have some delay until reach effective anti-viral activities, the innate immune responses might be crucial to reduce the viral replication and pathological alterations induced by a virus pathogen as well exert a relevant role in the activation and shaping of the anti-viral adaptive immune responses. Thus, we hypothesize that the suppressive effect on TLR7 pathway could be associated with an enhanced ability for viral replication and induction of lesions for both tracheal and renal tissues. However, further analyses of these assumptions are necessary.

In addition, a reduction of expression of CMI-genes for adaptive immune responses was also observed only in the group challenged with IBV B isolate, as the transcript levels of CD3 and CD4 were significantly downregulated at 1dpi only in this group. Granzyme homolog A and IFNγ transcripts were also differentially expressed between the challenged groups in this interval, as significant upregulation was observed only in A group. Therefore the downregulation of cell-mediated immune related genes could be a consequence of a primary virus challenge effect and could have negative consequences for the chicken host. In a previous study, we were able to detect an increase in the expression of cell-mediated immune related genes at 1 dpi in tracheal samples from birds vaccinated with Massachusetts attenuated IBV strain and challenged with a virulent homologous strain. This early CMI response, was associated with the level of cellular memory immune responses conferred by immunization, and this effect was dependent on the vaccine dose administered and was negative correlated with the tracheal pathological changes induced by M41 strain of IBV [8].

Although we have not evaluated in this study the gene expression and/or the biological activity of MHC I and II pathways, or other genes related to antigen presentation by dendritic cells, we can speculate that IBV B isolate might have some differentiated properties that confer to this virus a greater ability to decrease antigen presentation and to evade the host adaptive immune responses against this virus. This assumption is supported by the findings that all cell-mediated immune related genes analyzed here have unaltered expression or were significantly downregulated in the group infected by B isolate of IBV at 1dpi, as shown the significantly downregulated levels of expression observed for CD4 and CD3 transcripts, and unaltered expression for IFNγ and Granzyme homolog A genes in birds of this group. In contrast, at 1dpi, the ugroup challenged with IBV A isolate showed unaltered levels of CD3 and CD4 mRNA, while IFNγ and Granzyme homolog A transcripts were significantly upregulated.

Natural killer (NK) cells have important roles in the initial mechanisms of innate immunity against IBV, and were also important source of Granzyme homolog A and IFNγ. Although some authors have associated IFNγ production with the activity of NK cells after challenge with IBV [32], in our experiment, the profile of expression of IFNγ and Granzyme homolog A may as well be associated with T CD8+ activation, since CD8β and CD3 were also found to be upregulated in coincident time-points post-challenge in which IFNγ and Granzyme homolog A genes were upregulated, especially when considering that NK cells are unable to express CD8β and CD3 genes.

In summary, this study has contributed to the better understanding part of host-IBV interactions, and to our knowledge, it is the first study that demonstrate that expression of distinct innate and cell-mediated adaptive immune genes are induced by two IBV isolates that were classified in the same genotype but differing in their virulence activities. Overall, the results point out to the relevance of the involvement of the TLR7 pathway and the associate factors related to the suppression of early innate immune responses as well the early cell-mediated adaptive immune responses and the implications on the pathogenesis of infection by a more virulent IBV isolate. However, further studies are required to confirm this association, and to verify other cellular response pathways that may also be affected by this virus. It remains also to be further investigated the differences in the immune responses triggered by the IBV infection with more distinct genotypes of IBV strains that can differ in pathogenicity for the respiratory, urinary or other tissue targets for IBV infection in chickens. Moreover, it is important for poultry industry and indirectly for human health to better understand how coronaviruses infect the chicken host and which genes and/or pathways are effectively involved in increasing the severity of this disease, especially considering the diseases caused by other coronaviruses such as severe acute respiratory syndrome-associated coronavirus and Middle East respiratory syndrome coronaviruses.

## Supporting information

S1 File Protein alignment of S1 gene from A (BI/BR/Embrapa/331/2000) and B (BI/BR/Embrapa/127/2006) IBV isolates. Red squares are pointing the amino acids changes.
(DOCX)

S2 File Medians of Log10 IBV genome copies per interval per group, and c values for Kruskal Wallis test. (DOCX)

S3 File Medians of each treatment (gene expression) per interval per group, and c values for Kruskal Wallis test. (DOCX)

S4 File Comparison of Log10 IBV genome copies medians between groups per tissue per interval and P values by Wilcoxon test. (DOCX)

S5 File Comparison of gene expression medians between groups per treatment per interval and P values by Wilcoxon test. (DOCX)
