# Supplementary material for: Early immune responses and development of pathogenesis of avian infectious bronchitis viruses with different virulence profiles
Source: PLoS One. 2017 Feb 15;12(2):e0172275. doi: 10.1371/journal.pone.0172275 (PMC5310907; doi:10.1371/journal.pone.0172275)

**Protein alignment of S1 gene from A (BI/BR/Embrapa/331/2000) and B (BI/BR/Embrapa/127/2006) IBV isolates.** Red squares are pointing the amino acids changes.


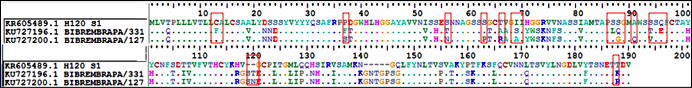

Supplement: S1 File — Red squares are pointing the amino acids changes. (DOCX) [file pone.0172275.s001.docx]
