# Supplementary material for: Early immune responses and development of pathogenesis of avian infectious bronchitis viruses with different virulence profiles
Source: PLoS One. 2017 Feb 15;12(2):e0172275. doi: 10.1371/journal.pone.0172275 (PMC5310907; doi:10.1371/journal.pone.0172275)
Supplement: S2 File — (DOCX) [file pone.0172275.s002.docx]

**Medians of Log10 IBV genome copies per interval per group, and c values for Kruskal Wallis test .**

|  |  | Log10 IBV genome copies (Median) per group | | |  |
| --- | --- | --- | --- | --- | --- |
| Tissue | Interval (dpi) | A | B | NC | cValue |
| Kidney | 5 | 3.651 | 3.686 | 0.000 | 0.0003 |
| Kidney | 8 | 0.000 | 5.452 | 0.000 | 0.0015 |
| Trachea | 1 | 5.757 | 7.875 | 0.000 | 0.0002 |
| Trachea | 5 | 6.263 | 7.613 | 0.000 | <.0001 |
| Trachea | 8 | 4.078 | 3.994 | 0.000 | 0.0065 |
