# Supplementary material for: Early immune responses and development of pathogenesis of avian infectious bronchitis viruses with different virulence profiles
Source: PLoS One. 2017 Feb 15;12(2):e0172275. doi: 10.1371/journal.pone.0172275 (PMC5310907; doi:10.1371/journal.pone.0172275)
Supplement: S3 File — (DOCX) [file pone.0172275.s003.docx]

**Medians of each treatment (gene expression) per interval per group, and c values for Kruskal Wallis test .**

|  |  | Fold change (Median) per group | | |  |
| --- | --- | --- | --- | --- | --- |
| Gene | Interval (dpi) | A | B | NC | cValue |
| CD3 | 1 | 0.986 | 0.353 | 1.178 | 0.0007 |
| CD3 | 5 | 10.7 | 12.2 | 1.168 | 0.0002 |
| CD3 | 8 | 11.3 | 10.0 | 1.233 | 0.0058 |
| CD4 | 1 | 1.158 | 0.476 | 0.917 | 0.0044 |
| CD4 | 5 | 9.092 | 13.5 | 1.160 | 0.0001 |
| CD4 | 8 | 5.029 | 4.216 | 0.947 | 0.0053 |
| CD8 | 1 | 1.194 | 1.148 | 0.911 | 0.4034 |
| CD8 | 5 | 19.6 | 17.2 | 1.308 | 0.0002 |
| CD8 | 8 | 9.903 | 10.0 | 0.982 | 0.0078 |
| GZHA | 1 | 2.074 | 1.085 | 0.981 | 0.0489 |
| GZHA | 5 | 67.2 | 101.0 | 0.936 | 0.0001 |
| GZHA | 8 | 90.1 | 94.6 | 1.163 | 0.0069 |
| IFNA | 1 | 1.307 | 1.778 | 1.350 | 0.4222 |
| IFNA | 5 | 3.093 | 3.192 | 1.026 | 0.0351 |
| IFNA | 8 | 2.926 | 2.258 | 1.245 | 0.0150 |
| IFNB | 1 | 1.020 | 32.8 | 1.709 | 0.0348 |
| IFNB | 5 | 7.941 | 19.3 | 1.106 | 0.0006 |
| IFNB | 8 | 4.176 | 3.172 | 0.769 | 0.0772 |
| IFNY | 1 | 2.909 | 2.836 | 0.974 | 0.0187 |
| IFNY | 5 | 25.9 | 51.3 | 1.208 | <.0001 |
| IFNY | 8 | 15.3 | 10.7 | 0.933 | 0.0076 |
| IL1B | 1 | 2.637 | 5.437 | 1.316 | 0.0020 |
| IL1B | 5 | 2.006 | 3.662 | 1.415 | 0.0057 |
| IL1B | 8 | 7.078 | 2.607 | 1.298 | 0.0064 |
| IL6 | 1 | 2.210 | 5.204 | 1.049 | 0.0148 |
| IL6 | 5 | 3.104 | 2.823 | 0.931 | 0.0002 |
| IL6 | 8 | 3.259 | 1.658 | 1.320 | 0.0671 |
| INOS | 1 | 5.012 | 5.209 | 1.397 | 0.0039 |
| INOS | 5 | 0.256 | 0.654 | 1.224 | 0.0767 |
| INOS | 8 | 0.451 | 0.430 | 0.816 | 0.3102 |
| MYD88 | 1 | 2.472 | 2.332 | 1.213 | 0.0009 |
| MYD88 | 5 | 2.143 | 2.885 | 1.076 | 0.0004 |
| MYD88 | 8 | 3.104 | 1.630 | 0.913 | 0.0038 |
| TLR3 | 1 | 8.148 | 10.5 | 1.134 | 0.0004 |
| TLR3 | 5 | 3.816 | 4.263 | 0.910 | 0.0001 |
| TLR3 | 8 | 3.499 | 1.807 | 1.031 | 0.0133 |
| TLR7 | 1 | 1.466 | 0.283 | 1.006 | 0.0005 |
| TLR7 | 5 | 4.207 | 4.730 | 1.201 | 0.0008 |
| TLR7 | 8 | 3.500 | 3.739 | 1.144 | 0.0077 |
| TNFSF15 | 1 | 0.937 | 0.671 | 1.081 | 0.2291 |
