# Supplementary material for: Early immune responses and development of pathogenesis of avian infectious bronchitis viruses with different virulence profiles
Source: PLoS One. 2017 Feb 15;12(2):e0172275. doi: 10.1371/journal.pone.0172275 (PMC5310907; doi:10.1371/journal.pone.0172275)
Supplement: S4 File — (DOCX) [file pone.0172275.s004.docx]

**Comparison of Log10 IBV genome copies medians between groups per tissue per interval and P values by Wilcoxon test.**

|  |  | Comparison between groups (P values) | | |
| --- | --- | --- | --- | --- |
| Tissue | Interval (dpi) |  | B | A |
| Kidney | 5 | A | 0.8946 |  |
| Kidney | 5 | NC | 0.0001 | 0.0005 |
| Kidney | 8 | A | 0.0041 |  |
| Kidney | 8 | NC | 0.0053 | 0.3613 |
| Trachea | 1 | A | 0.0543 |  |
| Trachea | 1 | NC | 0.0003 | 0.0012 |
| Trachea | 5 | A | 0.0041 |  |
| Trachea | 5 | NC | 0.0001 | 0.0001 |
| Trachea | 8 | A | 0.7150 |  |
| Trachea | 8 | NC | 0.0053 | 0.0041 |
