# Supplementary material for: Early immune responses and development of pathogenesis of avian infectious bronchitis viruses with different virulence profiles
Source: PLoS One. 2017 Feb 15;12(2):e0172275. doi: 10.1371/journal.pone.0172275 (PMC5310907; doi:10.1371/journal.pone.0172275)
Supplement: S5 File — (DOCX) [file pone.0172275.s005.docx]

**Comparison of gene expression medians between groups per treatment per interval and P values by Wilcoxon test.**

| Gene | Interval (dpi) | Comparison between groups (P values) | | |
| --- | --- | --- | --- | --- |
|  |  |  | B | A |
| CD3 | 1 | A | 0.0011 |  |
| CD3 | 1 | NC | 0.0015 | 0.6744 |
| CD3 | 5 | A | 0.8946 |  |
| CD3 | 5 | NC | 0.0003 | 0.0003 |
| CD3 | 8 | A | 0.2733 |  |
| CD3 | 8 | NC | 0.0090 | 0.0062 |
| CD4 | 1 | A | 0.0039 |  |
| CD4 | 1 | NC | 0.0071 | 0.7527 |
| CD4 | 5 | A | 0.4015 |  |
| CD4 | 5 | NC | 0.0003 | 0.0003 |
| CD4 | 8 | A | 0.2012 |  |
| CD4 | 8 | NC | 0.0090 | 0.0062 |
| CD8 | 1 | A | 0.7728 |  |
| CD8 | 1 | NC | 0.2110 | 0.2936 |
| CD8 | 5 | A | 0.9648 |  |
| CD8 | 5 | NC | 0.0003 | 0.0003 |
| CD8 | 8 | A | 1.0000 |  |
| CD8 | 8 | NC | 0.0090 | 0.0062 |
| GZHA | 1 | A | 0.0675 |  |
| GZHA | 1 | NC | 0.5637 | 0.0209 |
| GZHA | 5 | A | 0.2332 |  |
| GZHA | 5 | NC | 0.0003 | 0.0003 |
| GZHA | 8 | A | 0.4652 |  |
| GZHA | 8 | NC | 0.0090 | 0.0062 |
| IFNA | 1 | A | 0.1489 |  |
| IFNA | 1 | NC | 0.4414 | 0.5286 |
| IFNA | 5 | A | 0.8946 |  |
| IFNA | 5 | NC | 0.0118 | 0.0576 |
| IFNA | 8 | A | 0.7150 |  |
| IFNA | 8 | NC | 0.0163 | 0.0106 |
| IFNB | 1 | A | 0.0675 |  |
| IFNB | 1 | NC | 0.0093 | 0.8836 |
| IFNB | 5 | A | 0.1451 |  |
| IFNB | 5 | NC | 0.0005 | 0.0039 |
| IFNB | 8 | A | 0.8551 |  |
| IFNB | 8 | NC | 0.0526 | 0.0389 |
| IFNY | 1 | A | 0.5006 |  |
| IFNY | 1 | NC | 0.1489 | 0.0016 |
| IFNY | 5 | A | 0.0703 |  |
| IFNY | 5 | NC | 0.0003 | 0.0003 |
